# A Mobility Management Using Follow-Me Cloud-Cloudlet in Fog-Computing-Based RANs for Smart Cities

**DOI:** 10.3390/s18020489

**Published:** 2018-02-06

**Authors:** Yuh-Shyan Chen, Yi-Ting Tsai

**Affiliations:** Department of Computer Science and Information Engineering, National Taipei University, No. 151, University Rd., San Shia District, New Taipei City 23741, Taiwan; cherry21739@gmail.com

**Keywords:** smart city, follow-me edge, edge/fog computing, fog-computing-based RAN, mobility management

## Abstract

Mobility management for supporting the location tracking and location-based service (LBS) is an important issue of smart city by providing the means for the smooth transportation of people and goods. The mobility is useful to contribute the innovation in both public and private transportation infrastructures for smart cities. With the assistance of edge/fog computing, this paper presents a fully new mobility management using the proposed follow-me cloud-cloudlet (FMCL) approach in fog-computing-based radio access networks (Fog-RANs) for smart cities. The proposed follow-me cloud-cloudlet approach is an integration strategy of follow-me cloud (FMC) and follow-me edge (FME) (or called cloudlet). A user equipment (UE) receives the data, transmitted from original cloud, into the original edge cloud before the handover operation. After the handover operation, an UE searches for a new cloud, called as a migrated cloud, and a new edge cloud, called as a migrated edge cloud near to UE, where the remaining data is migrated from the original cloud to the migrated cloud and all the remaining data are received in the new edge cloud. Existing FMC results do not have the property of the VM migration between cloudlets for the purpose of reducing the transmission latency, and existing FME results do not keep the property of the service migration between data centers for reducing the transmission latency. Our proposed FMCL approach can simultaneously keep the VM migration between cloudlets and service migration between data centers to significantly reduce the transmission latency. The new proposed mobility management using FMCL approach aims to reduce the total transmission time if some data packets are pre-scheduled and pre-stored into the cache of cloudlet if UE is switching from the previous Fog-RAN to the serving Fog-RAN. To illustrate the performance achievement, the mathematical analysis and simulation results are examined in terms of the total transmission time, the throughput, the probability of packet loss, and the number of control messages.

## 1. Introduction

Cloud computing driving centralization is shown to be useful to lower the marginal costs of system administration and operations, and edge computing is a new technique for an alternative to cloud computing by moving the computing resources and analysis works from the cloud to the edge, referred to as cloudlet or fog nodes. The fog nodes are placed in close proximity to mobile devices to deliver highly response cloud services [1]. It is highly challenge to integrate the edge/fog computing techniques into smart IoT infrastructure for the smart city. A fog-computing-based radio access network, namely F-RAN, had proposed in [2] to take the advantage of local radio signal processing, cooperative radio resource management, and distributed storing capabilities in edge devices to significantly overcome the disadvantage of cloud-RAN [2] of the heavy burden on front-haul communications and the large amount of radio signal processing operations in the centralized baseband unit pool of cloud-RAN [2]. In an F-RAN, edge node is endowed with caching capabilities and is controllable from a central cloud processor as in a C-RAN [3] to reduce the delivery latency. Mobility management allows UEs or goods to move access multiple points while keeping their data sessions. Distributed mobility management (DMM) is proposed in [4] to distribute the mobility anchors in the data plane in flattening the mobility network such that the mobility anchors are positioned closer to UE [4]. Distributed mobility management for future 5G networks is emerging as a valid framework taking into account the requirements for large traffic in the core and the rise of extremely dense wireless access networks [5]. This work aims to develop a new DMM with low delivery latency using proposed Follow-Me Cloud-Cloudlet (FMCL) concept in F-RANs for the smart city. There is a novel and similar result in [6] to present a Fog-supported smart city network architecture called Fog Computing Architecture Network (FOCAN). To decrease latency and improve energy provisioning and the efficiency of services among things with different capabilities, the applications running on things jointly compute, route, and communicate with one another through the smart city environment.

Before describing the proposed follow-me cloud-cloudlet approach, the Follow-Me Cloud (FMC) and Follow-Me Edge concepts are initially described. Follow-Me Cloud (FMC) concept is proposed in [7] to migrate user service by virtual machines (VMs) between data centers (DCs) to support the service migration and continuity due to UE mobility or load balancing. Many mobile applications are heavily based on data and processing capabilities from the cloud [8]. Fog computing paradigm arises to overcome high delay encountered when real time applications need the low latency to access data or things of smart cities. Cloudlets allow the low latency access to data and processing capabilities, which can be accomplished by dynamically building a local virtual machine (VM) near to UEs or goods. A fog computing-based architecture in [8] supports the handoff by the local VM migration. In addition, Mobile Edge Computing (MEC) research is proposed by realizing the Follow-Me Edge (FME) concept [9] to ensure that the service constantly follows the user and that the user is always serviced from the closest edge [9].

It is observed that Follow-Me Cloud (FMC) results do not have the property of the VM migration between cloudlets for the purpose of reducing the transmission latency. In contrast, Follow-Me Edge (FME) results do not keep the advantage of the service migration between data centers (DCs) for reducing the transmission latency. Efforts will be made to propose a follow-me cloud-cloudlet (FMCL) approach to keep the VM migration between cloudlets and service migration between data centers (DCs) to significantly reduce the transmission latency. With the assistance of edge/fog computing, this paper presents a mobility management using a proposed follow-me cloud-cloudlet (FMCL) approach in fog-computing-based radio access networks (Fog-RANs) for smart cites. The proposed follow-me cloud-cloudlet approach is an integration strategy of follow-me cloud and follow-me edge. The new proposed mobility management aims to reduce the total transmission time if some data packets are pre-scheduled and pre-stored into the cache of cloudlet if UE is switching from the previous Fog-RAN to the serving Fog-RAN. The main contributions of the proposed handover scheme with FMCL approach are summarized as follows.

- We had proposed follow-me cloud-cloudlet (FMCL) approach, which is a integration strategy of follow-me cloud and follow-me edge to inherit the properties of FMC and FME and explore the cooperation between clouds and cloudlets.
- We had proposed a new mobility management with using FMCL approach to reduce the total transmission time, upgrade the throughput, and reduce the probability of the packet loss. This is because that the transmission cooperation between the cloud and the cloudlet, while some packets can be pre-scheduled in the cache of cloudlets to reduce the total transmission time and upgrade the throughput. This is because that some pre-scheduled packets can be directly accessed from local cloudlet, and these pre-scheduled packets are avoided the long network transmission to further improve the probability of the packet loss.

The rest of the paper is organized as follows. Section 2 describes the related work and motivation. Section 3 describes the system model, problem formulation and basic idea of our proposed scheme. Section 4 describes our proposed handover protocol with FMCL. Section 5 provides the performance analysis, and the conclusion is finally given in Section 6.

## 2. Related Works

This section first describes related work in Section 2.1 and then provides the research motivation in Section 2.2.

### 2.1. Related Works

The work mainly discusses the mobility management in the fog-computing-based RANs for smart city. There are some results for fog-computing-based RANs and some related handover works in [1,2,3,10,11,12,13]. Satyanarayanan et al. [1] had explained the background, effect, and application of the edge computing. Edge computing is a new paradigm in which substantial computing and storage resources—variously referred to as cloudlets, micro datacenters, or fog nodes—are placed at the Internet’s edge in close proximity to mobile devices or sensors [1]. Peng et al. [2] proposed some issues and challenges about fog-computing-based radio access networks. Peng et al. [2] declared that the fog-RAN-based architecture is a model for the 5-G mobile networks. The main idea includes local radio signal processing, cooperative radio resource management and distributed storing capabilities in edge devices. Tandon et al. [3] considered a hybrid architecture, referred to as fog RAN (F-RAN), and presented an information-theoretic framework. The information-theoretic framework characterized the main trade-offs between performance of an F-RAN, in terms of the worst case of the delivery latency and resources of caching and fronthaul capacities. Liang et al. [10] developed the offloading services from the cloud to the edge of networks in fog-computing-based platform for offering the real-time data services to the nearby data terminal. Tandon et al. [11] defined the fog radio access network (Fog-RAN) architecture in [11]. Fog-RAN is an emerging wireless network architecture. This architecture utilizes the caching capabilities at the edge nodes to provide a low data access latency. Jalali et al. [12] studied about the energy consumption problem of nano data centers (nDCs) for the edge computing data center. Shin et al. [13] introduced the Fog-Radio Access Network (F-RAN) architecture to bring the efficient computing capability of the cloud to the network edge. By distributing computing-intensive tasks to multiple F-RAN nodes, F-RAN has the potential to meet the requirements of those ultra low-latency applications.

Some results of distributed mobility managements are reported in the literature. For instance, Balfaqih et al. [14] presented a network-based DMM solution. This work also developed an analytical model to evaluate the handover latency and the packet loss. To improve the performance during the handover, this work specifies a buffer technique at the new Mobile Anchor Access Router (nMAAR) for the packet buffering. Murtadha et al. [15] also designed a network-based fully distributed mobility management in flattened network architecture. In addition, some SDN-based mobility managements are recently reported in [16,17,18,19,20,21,22,23]. One interest result is a SDN-based handover result reported in [16,17]. Modares et al. [16] provides a useful survey on proxy mobile IPv6 hsndover. Raza et al. [17] proposed an inter-domain IP mobility solution with route optimization using the SDN-based proxy mobile IPv6. Wang et al. [18] focused on extending SDN paradigm to mobility handling in the Internet, and to propose the design, implementation and deployment of a software-defined IP mobility architecture. Yao et al. [19] proposed a dynamic switch migration scheme by dynamically adapting the the data flow to realize the load balance among multiple SDN controller. Garzon et al. [20] proposed an X2-based handover procedure in an SDN-based LTE architecture. Elgendi et al. [22] proposed a distributed mobility management (DMM) and a user rate-perceived (URP) algorithm over a 3-Tier SDN-based architecture. Carpio et al. [23] proposed a new load balancing solution in SDN-based data center networks.

Mobility management results using the concept of follow-me cloud (FMC) are recently studied in [7,24,25,26,27]. Aissioui et al. [24] proposed proxy MIPv6-based FMC in [24] by a inter-domain distributed mobility management. Nadembega et al. [25] proposed a mobility-based services migration prediction (MSMP) scheme by addressing the trade-off between the overhead and Quality of Experience (QoE). Ksentini et al. [26] further considered the trade-off by modeling the service migration issue by using a Markov Decision Process (MDP).

Mobility management results using the concept of follow-me edge (FME) is studided in [9]. Taleb et al. [9] realized the FME concept by enforcing an autonomic creation of Mobile Edge Computing (MEC) service to allow the data access with the optimum QoE and the reduced latency. To realize the FME, the Edge Orchestrator (EO) of the mobile network operators (MNOs) needs to update the resource information and the user location information.

Some novel results of cloud and fog services to vehicular networks had developed [28,29]. Shojafar et al. [28] proposed an energy-efficient adaptive resource management for real-time vehicular cloud services. Naranjo et al. [29] proposed an energy-efficient adaptive scheduler for Vehicular Fog Computing (VFC) that operates at the edge of a vehicular network. Some results of 5G mobile networks with caching are provided [30,31,32,33]. Zakrzewska et al. [30] investigated the key challenges and current trends of 5G mobile networks.

Park et al. [31] developed a joint optimization of cloud and edge processing for fog radio access networks. Peng et al. [32] developed a backhaul-aware caching placement for wireless networks. Ugur et al. [33] developed a cloud radio access networks with coded caching.

### 2.2. Motivation

As mentioned in Section 2.1, two kinds of mobility managements are reported; one is the mobility managements using the concept of follow-me cloud (FMC) [7,24,25,26,27] and another one is the mobility management using the concept of follow-me edge/cloudlet (FME) [9]. The main motivation of this work is attempted to propose a new mobility management with the integration of the concepts of follow-me cloud (FMC) [7,24,25,26,27] and the follow-me edge/cloudlet (FME) [9], which is called as follow-me cloud-cloudlet (FMCL) approach in this paper. Existing FMC results do not own the novel property of the VM migration between cloudlets for the purpose of reducing the transmission latency. All existing (FME) results do not keep the advantage of the service migration between data centers (DCs) for reducing the transmission latency. Efforts will be made to propose a follow-me cloud-cloudlet (FMCL) approach to keep the VM migration between cloudlets and service migration between data centers (DCs) to significantly reduce the transmission latency. Thus, this work is to propose a new mobility management using the FMCL approach for the smart cities. One interested capability of fog-computing-based RAN architecture is that F-AP (fog-computing-based access point) of F-RAN contains the local cache functionality. Consequently, the new mobility management with the FMCL approach can inherit the advantage of the service migration from follow-me cloud (FMC) [7,24,25,26,27] and can explore and additionally keep the data locality property from the local cache functionality from the follow-me edge (FME) [9].

Consequently, one of the benefits is that the optimal shortest route can be re-calculated and re-constructed from the new service migrated data center to significantly reduce the network transmission latency. Another one is that all received packets can be pre-scheduled to the cache of edge/cloudlet to keep this data locality property of cache to improve the throughput and reduce the packet loss rate.

## 3. Preliminaries

This section describes the system model, the problem formulation, and basic idea in Section 3.1, Section 3.2 and Section 3.3.

### 3.1. System Architecture

**Figure 1:**
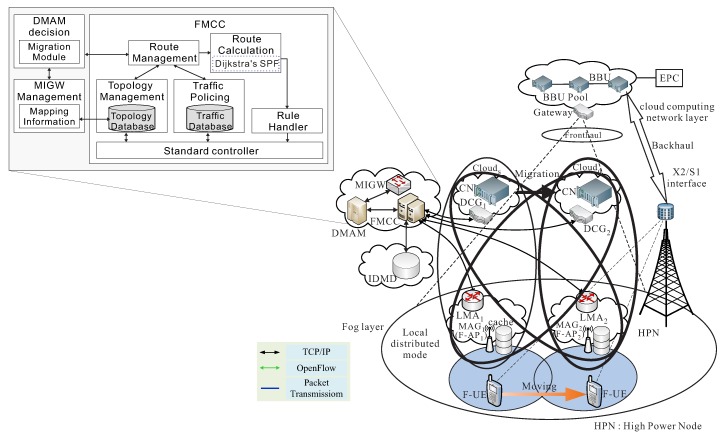
System architecture.

The fog-computing-based RAN system architecture evolution from cloud-radio access network (C-RAN) [1,2,3] is shown in Figure 1. To overcome the disadvantages of C-RANs with the fronthaul constraints, the user and control planes are decoupled in such networks and the high power nodes (HPNs) are mainly used to provide seamless coverage and execute the functions of the control plane, while remote radio heads (RRHs) are deployed to provide high-speed data rate for packet traffic transmission in the user plane. The fronthaul portion of a C-RAN telecommunications architecture comprising the intermediate links between the centralized radio controllers and remote radio heads (RRHs) at the edge of a cellular network [1]. One main difference between C-RANs and F-RANs is that the centralized control function is shifted from the BBU pool in C-RANs to the HPN in F-RANs [2]. To incorporate fog computing in edge devices, the traditional RRH is evolved to the fog-computing-based access point (F-AP) by being equipped with a certain caching, CRSP, and CRRM capabilities [2]. The main difference between C-RANs and F-RANs is that the centralized control function is shifted from the BBU pool in C-RANs to the HPN in F-RANs [2].

**Figure 2:**
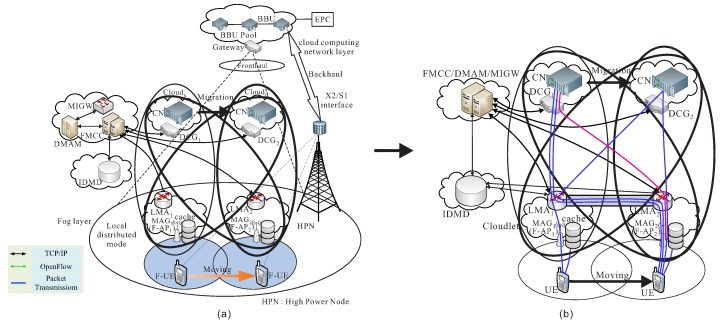
(**a**) Fog-RAN architecture using follow me cloud-cloudlet and (**b**) its simplified architecture.

As shown in Figure 2a, the system architecture for our proposed follow-me cloud-cloudlet is modified from the system architecture of mobility using follow-me cloud approach from Aissioui et al.’s work in [24], and its simplified architecture is given in Figure 2b. Consequently, our system architecture also contains the follow-me cloud controller (FMCC), decision making application module (DMAM), mapping information gateway (MIGW), inter domain mobility database (IDMD), and local mobility anchor (LMA) [24]. The service migration of follow-me cloud approach [24] is also provided and reviewed as follows. When an UE switches to a different region, FMCC then decides to initiate a service migration from the current serving data center to the new serving data center, and also initiate a handover procedure and re-calculate the shortest route from the new serving data center gateway (DCG) to the next LMA of the new serving F-AP. In our follow-me cloud-cloudlet approach, FMCC also pre-sends some packets to the cache of the new serving F-AP to reduce the total data transmission time.

In the paper, we propose a new mobility management using the follow-me cloud-cloudlet (FMCL) approach for the smart city. This main purpose of FMCL approach is to reduce the total transmission time and the packet loss rate, under a UE handover to a different area.

The network architecture of the fog-computing-based RANs (or called as F-RANs) contains that there is a cloud set $C=\left\{c_{1},c_{2},\cdots ,c_{s},\cdots ,c_{d},\cdots ,c_{n}\right\}$, where $c_{s}$ is the *s*-th serving data center or the *s*-th serving cloud, and cloudlet set $L=\left\{l_{1},l_{2},\cdots ,l_{s},\cdots ,l_{d},\cdots ,l_{n}\right\}$, where $l_{s}$ is the *s*-th serving cloudlet, then a cloud-cloudlet (CL) set is defined as $CL=\left\{c_{1}\to l_{1},c_{2}\to l_{2},\cdots ,c_{s}\to l_{d},\cdots ,c_{d}\to l_{s},\cdots ,c_{n}\to l_{n},\right\}$. In general, we assumed that an UE in the area with $l_{s}$ moves to a different area with $l_{d}$. Let $c_{s}\to l_{d}$ represent as the packet transmission from cloud $c_{s}$ to cloudlet $l_{d}$ during our mobility management using the cooperation transmission of cloud-cloudlet.

When the UE moves from a previous area with $l_{s}$ to a different area with $l_{d}$, the current serving $c_{s}$ is performed the service migration operation to the new serving $c_{d}$. The MAG of $l_{d}$ sends out a proxy binding update (PBU), $PBU\_message(UE\_id,prefix_{d},LMA_{d},TIM)$, to the inter domain mobility database (IDMD), where TIM (Traffic Information Message) is utilized to keep the receiving packet status of all packet transmissions during the handover procedure. The IDMD then creates an entry in its Binding Cache Entry (BCE) table. The new record of the BCE table is $BCE\_table(UE\_id,prefix_{d},LMA_{d})$. After the IDMD receiving PBU_message, the IDMD sends a migration-request message to the FMCC. The migration-request message contains the ID of UE, the allocated prefix, the new allocated LMA, and the cache data in $l_{d}$. After the FMCC obtaining the message, the FMCC concurrently finds the optimal transmission path between the new Data Center Gateway (DCG) of $c_{d}$ and new allocated LMA of $l_{d}$. When the FMCC determines the optimal path from $c_{d}$ to $l_{d}$, the FMCC sends out $REQ\_message(UE\_id,prefix_{s},prefix_{d},TIM)$ to the new $c_{d}$ to switch to the new path.

### 3.2. Problem Formulation

In the work, we attempted to develop a follow-me cloud-cloudlet approach for the mobility management. The main purpose is to reduce the transmission time during the handover by increasing the cache hit rate. As defined previously, The proposed follow-me cloud-cloudlet architecture contains a cloud-cloudlet (CL) set as $CL=\left\{c_{1}\to l_{1},c_{2}\to l_{2},\cdots ,c_{s}\to l_{d},\cdots ,c_{d}\to l_{s},\cdots ,c_{n}\to l_{n}\right\}$, where $l_{s}$, $l_{d}$, $c_{s}$, and $c_{d}$ are determined by FMCC during the handover operation. When FMCC decides the $l_{d}$, we try to pre-cache of the $l_{d}$ of the required data packets. Let α be denoted as the packet quantity existed in cache of the fog-computing-based RAN architecture. A data file *F* considered in this work is divided into *n* packets, $F=\left\{p_{1},p_{2},\cdots ,p_{s},\cdots ,p_{s},\cdots ,p_{d}\right\}$, where *n* is the total packet number of *F*. In our mobility protocol design, the total data transmission time when switching to different access point is $T_{FM}\left(p_{s}\right)$ + $T_{FML}$ + $T_{FMC}$ + $T_{FMCL}\left(p_{d}\right)$, where $T_{FM}\left(p_{s}\right)$ is the time cost of the *follow me* (FM) phase, $T_{FML}$ is the time cost of the *follow me cloudlet* (FML) phase $T_{FMC}\left(p_{d}\right)$ is the time cost of the *follow me cloud* (FMC) phase, and $T_{FMCL}\left(p_{s}\right)$ is the time cost of the *follow me cloud-cloudlet* (FMCL) phase (as described below). The problem is formulated to minimize the total data transmission time as:

(1) $$\begin{array}{c} \mathrm{minimize}\;\;\{\min\;\sum_{1\leq s\leq k}T_{FM}\left(p_{s}\right)+T_{FML}+T_{FMC}+\min\;\sum_{k\leq d\leq n}T_{FMCL}\left(p_{d}\right)\}\\ \mathrm{subject}\;\mathrm{to}\;\left\{\begin{array}{c} T_{FM}\left(p_{s}\right)=\;\;\;\left\{\begin{array}{c} \begin{array}{c} T_{c},\mathrm{if}\;p_{s}\;\in \;l_{s}\\ T_{d},\mathrm{otherwise}.\; \end{array} \end{array}\right.\\ \begin{array}{c} T_{FMCL}\left(p_{d}\right)=\left\{\begin{array}{c} \begin{array}{c} T_{c}^{\prime },\mathrm{if}\;p_{d}\;\in \;l_{d}\\ T_{d}^{\prime },\mathrm{otherwise},\; \end{array} \end{array}\right. \end{array} \end{array}\right. \end{array}$$

where $T_{c}<<T_{d}$, $T_{c}^{\prime }<<T_{d}^{\prime }$. Time $T_{c}$ and $T_{c}^{\prime }$ denoted that the data transmission time of packet $p_{s}$ and $p_{d}$ is directly accessed from cache of clodlets $l_{s}$ and $l_{d}$, $T_{d}$ and $T_{d}^{\prime }$ denoted that the data transmission time of packet $p_{s}$ and $p_{d}$ must be transmitted from data center of clouds $c_{s}$ and $c_{d}$, respectively.

In addition, Wang et al. [34] generally discussed two kinds of caching techniques; web caching and redundancy elimination (RE). There are three types of RE technique; chunk-level RE, TCP-level RE and the packet-level RE. In the paper, we adopted the packet-level RE as our caching technique [34].

### 3.3. Basic Idea

This basic idea of the mobility management is to propose a cooperation strategy of cloud and cloudlet to reduce the transmission time and the packet loss rate.

**Figure 3:**
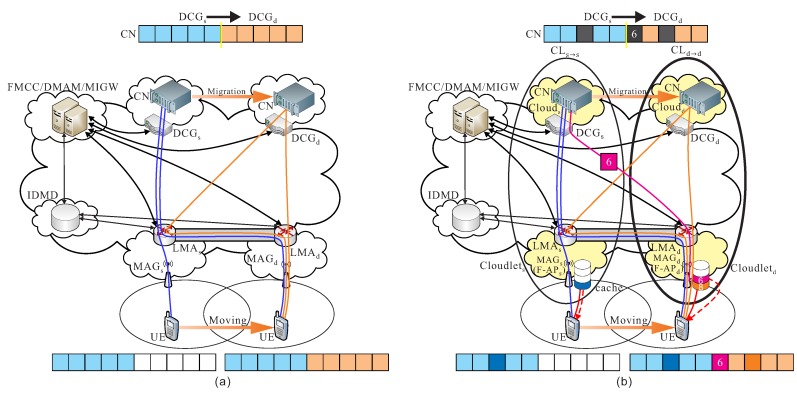
Comparison of (**a**) PMIPv6-based follow me cloud and (**b**) our proposed scheme using follow me cloud-cloudlet.

Based on the proxy MIPv6-based FMC result of [24], all data packets are tunneled between MAG (Mobile Access Gateway) and LMA (Local Mobility Anchor) to keep the high connectivity property. As mentioned before, a cloud-cloudlet (CL) set is $CL=\left\{c_{1}\to l_{1},c_{2}\to l_{2},\cdots ,c_{s}\to l_{d},\cdots ,c_{d}\to l_{s},\cdots ,c_{n}\to l_{n}\right\}$. When UE attached to a different MAG, four kinds of cooperation of cloud-cloudlet ($CL_{s\to s}$), cloud-cloudlet ($CL_{s\to d}$), cloud-cloudlet ($CL_{d\to s}$), and cloud-cloudlet ($CL_{d\to d}$) are defined below. The comparison of of proxy MIPv6-based FMC proposed by Aissioui et al. [24] and our the handover protocol using follow me cloud-cloudlet is illustrated in Figure 3.

**Figure 4:**
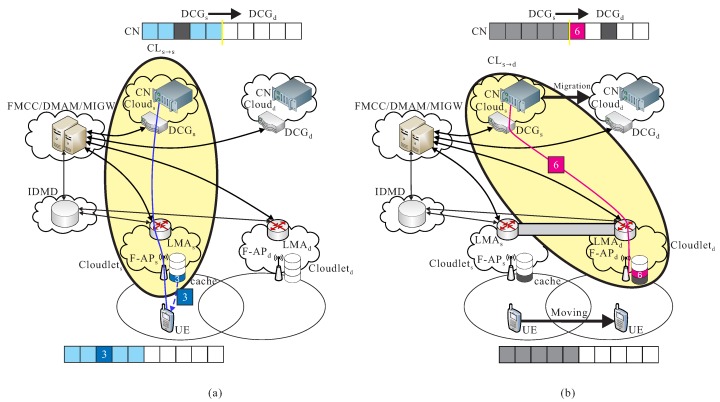
Basic operations of (**a**) cooperation of cloud-cloudlet $CL_{s\to s}$ and (**b**) cooperation of cloud-cloudlet $CL_{s\to d}$.

**Cooperation of cloud-cloudlet** $CL_{s\to s}$: Before the handover event of UE, assumed that $c_{s}$ and $l_{s}$ are the current serving cloud and cloudlet of the current serving F-RAN. Packets $p_{s}$ from the CN in data center of $c_{s}$ are transmitted to UE through F-AP${}_{s}$. For instance as shown in Figure 4a, the cooperation of cloud-cloudlet $CL_{s\to s}$ is provided.

**Cooperation of cloud-cloudlet** $CL_{s\to d}$: When UE moves to a different region, and initiate the handover procedure, some packets are still transmitted from the current serving data center of $c_{s}$ to the new serving cloudlet $l_{d}$ if the data migration procedure is still not initiated. For instance as shown in Figure 4b, the cooperation of cloud-cloudlet $CL_{s\to d}$ is provided.

**Figure 5:**
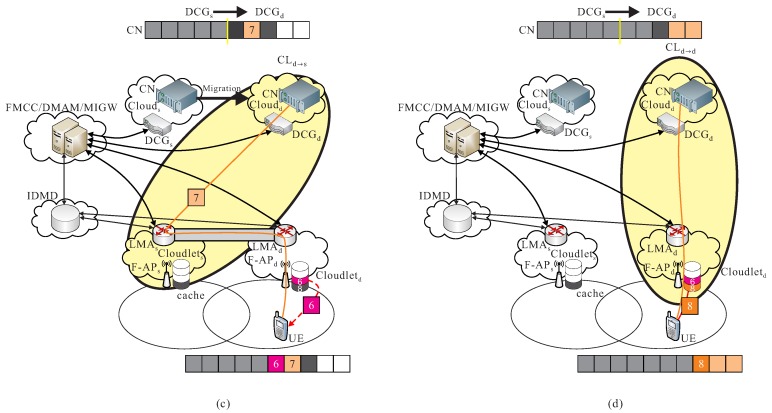
Basic operations of (**a**) cooperation of cloud-cloudlet $CL_{d\to s}$ and (**b**) cooperation of cloud-cloudlet $CL_{d\to d}$.

**Cooperation of cloud-cloudlet** $CL_{d\to s}$: After UE is moving to a different region, a data migration operation is executed from $c_{s}$ to $c_{d}$, it also means that CN is migrated from $c_{s}$ to $c_{d}$. Packets are then transmitted from the new serving data center of $c_{d}$ to the previous cloudlet $l_{s}$, and then be re-forward to the new cloudlet $l_{d}$ to UE, before the new route path from $c_{d}$ to $l_{d}$ is not re-calculated in FMCC. For instance as shown in Figure 5a, the cooperation of cloud-cloudlet $CL_{d\to s}$ is provided.

**Cooperation of cloud-cloudlet** $CL_{d\to d}$: After the new route path is re-routed from the new serving data center of $c_{d}$ to the new cloudlet $l_{d}$ which is determined by FMCC, packets $p_{d}$ are transmitted from $c_{d}$ to UE through F-AP${}_{d}$ of $l_{d}$ by using the new re-calculated route path. As shown in Figure 5b, an example of the cooperation of cloud-cloudlet $CL_{d\to d}$ is given.

## 4. Mobility Management Using Follow-Me Cloud-Cloudlet Approach

In the section, mobility management using proposed follow-me cloud-cloudlet approach in F-RAN for smart cities is given. As mentioned in Section 3.3, four kinds of cooperation of cloud-cloudlet are defined, our mobility management is divided into four phases to implement these four cooperation of cloud-cloudlet, respectively.

1. *Follow me phase*: This phase is to implement the cooperation of cloud-cloudlet $CL_{s\to s}$. Before the handover event of UE, assumed that $c_{s}$ and $l_{s}$ are the current serving cloud and cloudlet of the current serving F-RAN. Packets $p_{s}$ from the CN in data center of $c_{s}$ are transmitted to UE through F-AP${}_{s}$.
2. *Follow me cloudlet phase*: This phase is to implement the cooperation of cloud-cloudlet $CL_{s\to d}$. When UE moves to a different region, and initiate the handover procedure, some packets are still transmitted from the current serving data center of $c_{s}$ to the new serving cloudlet $l_{d}$ if the data migration procedure is still not initiated.
3. *Follow me cloud phase*: This phase is to implement the cooperation of cloud-cloudlet $CL_{d\to d}$. After UE is moving to a different region, a data migration operation is executed from $c_{s}$ to $c_{d}$, it also means that CN is migrated from $c_{s}$ to $c_{d}$. Packets are then transmitted from the new serving data center of $c_{d}$ to the previous cloudlet $l_{s}$, and then be re-forward to the new cloudlet $l_{d}$ to UE, before the new route path from $c_{d}$ to $l_{d}$ is not re-calculated in FMCC.
4. *Follow me cloud-cloudlet phase*: The phase is to implement the cooperation of cloud-cloudlet $CL_{d\to d}$. After the new route path is re-routed from the new serving data center of $c_{d}$ to the new cloudlet $l_{d}$ which is determined by FMCC, packets $p_{d}$ are transmitted from $c_{d}$ to UE through F-AP${}_{d}$ of $l_{d}$ by using the new re-calculated route path.

**Figure 6:**
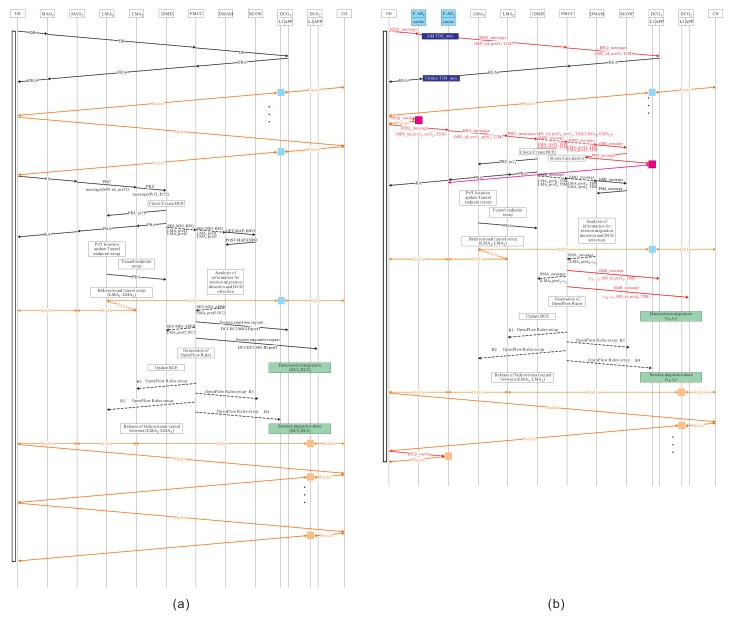
Message flow diagrams of (**a**) PMIPv6-based FMC approach and (**b**) our proposed mobility management using FMCL approach.

The detailed operations of the mobility management using proposed follow-me cloud-cloudlet approach in F-RAN are described as follows. Message flow diagrams of PMIPv6-based FMC approach and our proposed mobility management using FMCL approach, are illustrated in Figure 6.

### 4.1. Follow-Me Phase

This phase mainly implements the cooperation of cloud-cloudlet $CL_{s\to s}$. Let REQ_message $\left(UE\_id,prefix_{s},prefix_{d},TIM\right)$ or simplified as REQ_message is denoted as a data transmission request message, where UE_id is UE’s ID, $prefix_{s}$ and $prefix_{d}$ are UE’s the prefix of the network address, and TIM is the traffic indication map to indicate the packet receiving status.

Assumed that a data file is divided into *n* packets, $F=\left\{p_{1},p_{2},\cdots ,p_{n}\right\}$. TIM of REQ_message is *n*-bit map, $TIM=\left\{b_{1},b_{2},\cdots ,b_{k},\cdots ,b_{n}\right\}$, if $b_{k}$ is equal to 1 then *k*-th packet is received by UE, and if $b_{k}$ is equal to 0, then *k*-th packet is not received by UE, where 1≤k≤n. Before the handover event of UE, assume that $c_{s}$ and $l_{s}$ are the current serving cloud and cloudlet of the current serving F-RAN. Packets $p_{s}$ from the CN in data center of $c_{s}$ are transmitted or not, which is dependent on the TIM which is extracted from received $REQ\_message(UE\_id,prefix_{s},TIM)$, to UE through F-AP${}_{s}$.

The $REQ\_message(UE\_id,prefix_{s},TIM)$ message is initiated from UE and forward the REQ_message through the serving LMA to the inter domain mobility database (IDMD). After IDMD receiving REQ_message, a registration operation is executed in IDMD to create an entry in IDMD table as $UE\_information(UE\_id,prefix_{s},LMA_{s})$. The $REQ\_message(UE\_id,prefix_{s},TIM)$ is forward to follow me cloud controller (FMCC). if FMCC receives the message, $REQ\_message(UE\_id,prefix_{s},TIM)$ is also forward to to the serving data center (DC) of cloud $c_{s}$. DC of $c_{s}$ extracts $TIM=\left\{b_{1},b_{2},\cdots ,b_{k},\cdots ,b_{n}\right\}$ from received REQ_message. DC examines $TIM=\left\{b_{1},b_{2},\cdots ,b_{k},\cdots ,b_{n}\right\}$, if the value of $b_{k}$ of TIM_message is 0, then DC transmits *k*-packet toward UE through $l_{s}$. *UE* also keeps a same $TIM\_UE=\left\{b_{1},b_{2},\cdots ,b_{k},\cdots ,b_{n}\right\}$. UE changes the value of $b_{k}$ from 0 to 1 if UE receives *k*-th packet from the serving DC. Then, $TIM=(\underset{m}{\underset{︸}{1,1,\cdots ,1}}\underset{n-m}{\underset{︸}{0,0,\cdots ,0}})$. The detailed procedure is given below.

**S1.**  UE initiates $REQ\_message(UE\_id,prefix_{s},TIM)$, to F-AP, where $TIM=\left\{b_{1},b_{2},\cdots ,b_{k},\cdots ,b_{n}\right\}$, and $b_{k}$ =0, 1≤k≤n,
  (2) $$\begin{array}{c} TIM=\underset{n}{\underset{︸}{\left(0,0,\cdots ,0\right)}} \end{array}$$
**S2.**  The $REQ\_message(UE\_id,prefix_{s},TIM)$ reaches to F-AP${}_{s}$ of $l_{s}$. The F-AP${}_{s}$ checks if *k*-th packets is already existed in cache of F-AP${}_{s}$, then update $b_{k}$ of TIM accordingly, where 1≤k≤n. The updated TIM is re-inserted into the $REQ\_message(UE\_id,prefix_{s},$ updated TIM), and then forward the new REQ_message to IDMD and FMCC.
**S3.**  DC of $c_{s}$ extracts $TIM=\left\{b_{1},b_{2},\cdots ,b_{k},\cdots ,b_{n}\right\}$ from received REQ_message. Before the handover event, DC repeatedly examines $TIM=\left\{b_{1},b_{2},\cdots ,b_{k},\cdots ,b_{n}\right\}$, if the value of $b_{k}$, for 1≤k≤n, of TIM_message is 0, then serving DC transmits *k*-packet toward UE through $l_{s}$. *UE* also keeps $TIM\_UE=\left\{b_{1},b_{2},\cdots ,b_{k},\cdots ,b_{n}\right\}$, and UE concurrently updates the value of $b_{k}$ from 0 to 1 if UE successfully receives *k*-th packet.
**S4.**  If the handover decision of UE is made by switching from F_AP${}_{s}$ to F_AP${}_{d}$, then go to the *follow-me cloudlet* phase. Then, $TIM=(\underset{m}{\underset{︸}{1,1,\cdots ,1}}\underset{n-m}{\underset{︸}{0,0,\cdots ,0}})$.

**Figure 7:**
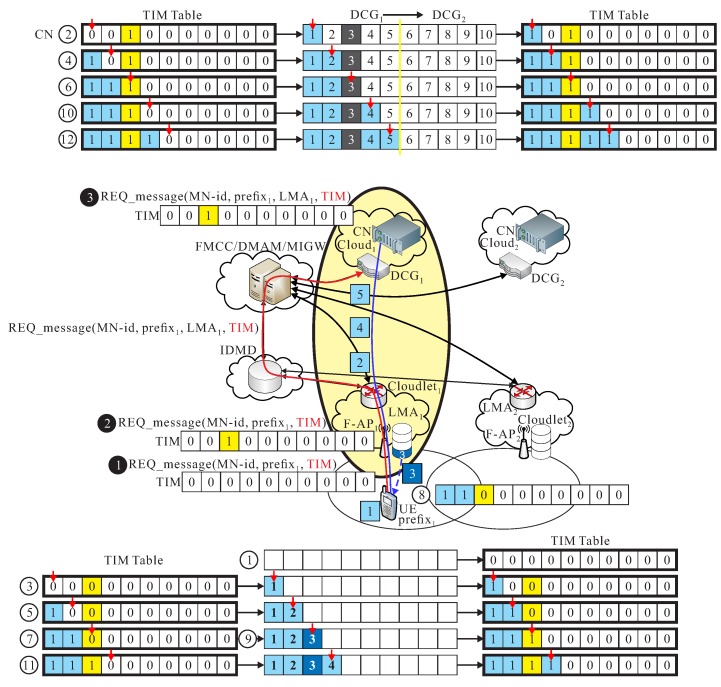
An example of *follow me* phase.

As shown in Figure 7, UE sends out $REQ\_message(UE\_id,prefix_{1},TIM)$ with TIM=(0,0,1,0,0,0,0,0,0,0) updated by F${}_{1}$ through IDMD to FMCC, because that 3-th packet existed is in cache of $l_{1}$. Finally, packets 1, 2, 3, and 4 are successfully received by UE, but packets 1, 2, 4, and 5 are transmitted from $c_{1}$. Therefore, TIM_UE = (1, 1, 1, 1, 0, 0, 0, 0, 0, 0).

### 4.2. Follow-Me Cloudlet Phase

This phase implements the cooperation of cloud-cloudlet $CL_{s\to d}$. After performing *follow-me* phase, assume that we have $TIM=(\underset{m}{\underset{︸}{1,1,\cdots ,1}}\underset{n-m}{\underset{︸}{0,0,\cdots ,0}})$. When UE moves to a different region, and initiate the handover procedure, un-transmitted packets are still transmitted from the current serving data center of $c_{s}$ to the new serving cloudlet $l_{d}$, note that the data migration procedure is still not initiated, such that we may have $TIM=(\underset{m^{\prime }}{\underset{︸}{1,1,\cdots ,1}}\underset{n-m^{\prime }}{\underset{︸}{0,0,\cdots ,0}})$, where $m\leq m^{\prime }$. That is, there are $m^{\prime }-m$ pre-transmitted packets. Some of these $n-m^{\prime }$ bits of TIM can be set to be 1 if these corresponding packets are already exited in cache of $l_{d}$.

The detailed procedure is given.

**S1.**  When UE is moving to the new region with F-AP${}_{d}$ of $l_{d}$, the UE initiates a request message, namely $REQ\_message(UE\_id,prefix_{s},prefix_{d},TIM)$, if the TIM message indicates that *m* packets are already successfully received by UE after executing the follow-me phase. The REQ_message also informs FMCC to carry the handover information with the report of the remaining un-received packets. The F-AP${}_{d}$ also checks if *k*-th packets is already existed in cache of F-AP_*s*_, then let $b_{k}=1$ of TIM , where m+1≤k≤n. The updated TIM is re-inserted into the $REQ\_message(UE\_id,prefix_{s},$ updated TIM) and forward to IDMD and FMCC.
**S2.**  MAG in $l_{d}$ receives the $REQ\_message(UE\_id,prefix_{s},$TIM) from F-AP${}_{d}$, MAG initiates a proxy binding update (PBU), $PBU\_message(UE\_id,prefix_{s},prefix_{d},TIM)$ or PBU_message to LMA and forward it to IDMD. After IDMD receiving PBU_message, IDMD updates the binding cache entry (BCE) table, $BCE\_table[UE\_id,prefix_{s},prefix_{d},LMA_{s},LMA_{d}]$, BCE_table. The IDMD generates the proxy binding acknowledgement (PBA), PBA_message to two LMA of the $l_{s}$ and $l_{d}$.
**S3.**  FMCC receives the *session-migration-request* message, $SMR\_message(MN\_id,prefix_{s},prefix_{d})$ from IDMD if IDMD receives $REQ\_message(UE\_id,prefix_{s},$ updated TIM). FMCC sends $SMR\_message(MN\_id,prefix_{s},prefix_{d})$ to decision making application module (DMAM). DMAM is activated by the request from FMCC. DMAM is responsible of making the decision of the data migration to search for an optimal cloud $c_{d}$.
**S4.**  DMAM analyzes the user information, $UE\_information(prefix_{s},prefix_{d},etc.)$ in addition to the mapping information of $c_{d}$ and $l_{d}$ by generating *get-mapping-information* message, GMI_message, to mapping information gateway (MIGW). MIGW then initiates a post-mapping-information message, PMI_message, to DMAM.
**S5.**  After DC of cloud $c_{s}$ verifying the received TIM message, the DC of cloud $c_{s}$ randomly pre-transmits some $k^{\prime }$-th un-transmitted packets, where $b_{k}^{\prime }=0$ and $m+1\leq k^{\prime }\leq n$. The corresponding bits are set 1, for $b_{k}^{\prime }=1$, in the TIM message if the packet are successfully pre-transmitted toward to cloudlet $l_{d}$ and kept the pre-transmitted packets in cache of $l_{d}$. This pre-transmission operation is done until the new route path is determined in *follow-me cloud-cloudlet* phase. Finally, some of these $n-m^{\prime }$ bits of TIM can be set to be 1 if these corresponding packets are already exited in cache of $l_{d}$.
**S6.**  Finally, FMCC notifies $c_{s}$ and $c_{d}$ about the current user’s information containing current TIM message, the location information through the analysis of DMAM and MIGW.

**Figure 8:**
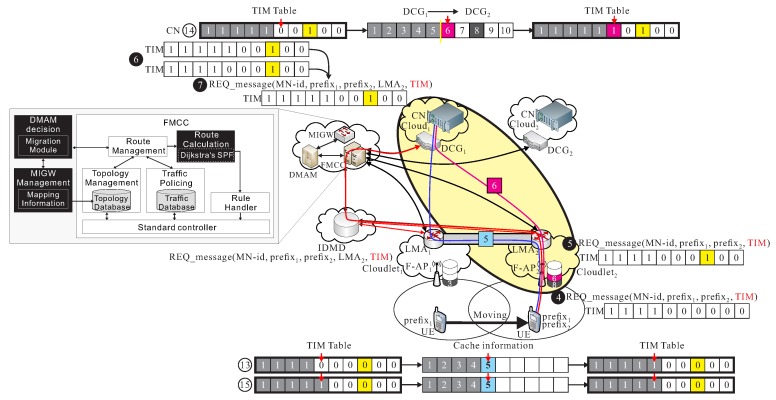
An example of *follow me cloudlet* phase.

As shown in Figure 8, UE moves from $l_{1}$ to $l_{2}$ and transmits a new $REQ\_message(UE\_id,prefix_{1},prefix_{2},TIM)$ to $l_{2}$, where TIM is (1, 1, 1, 1, 0, 0, 0, 0, 0, 0). This phase allows 5-th packet can be re-forward from $l_{1}$ to $l_{2}$, and allows 6-th packet can be pre-transmitted from $c_{1}$ to $l_{2}$, so TIM becomes (1, 1, 1, 1, 1, 0, 0, 0, 0, 0), but 6-th packet is pre-transmitted, but, finally TIM = (1, 1, 1, 1, 1, 0, 0, 1, 0, 0), since we assumed 7-th packet is already existed in cache of $l_{2}$.

### 4.3. Follow-Me Cloud Phase

This phase mainly implements the cooperation of cloud-cloudlet $CL_{d\to d}$. After UE is moving to a different region, a data migration operation is executed from $c_{s}$ to $c_{d}$, it also means that CN is migrated from $c_{s}$ to $c_{d}$. After performing *follow-me cloudlet* phase, assume that we have $TIM=(\underset{m^{\prime }}{\underset{︸}{1,1,\cdots ,1}}\underset{n-m^{\prime }}{\underset{︸}{0,0,\cdots ,0}})$. Thus, $m^{\prime \prime }-m^{\prime }$ packets are then transmitted from the new serving data center of $c_{d}$ to the previous cloudlet $l_{s}$, and then be re-forward to the new cloudlet $l_{d}$ to UE, before the new route path from $c_{d}$ to $l_{d}$ is not re-calculated in FMCC. Then, we have $TIM=(\underset{m^{\prime \prime }}{\underset{︸}{1,1,\cdots ,1}}\underset{n-m^{\prime \prime }}{\underset{︸}{0,0,\cdots ,0}})$, where $m^{\prime }\leq m^{\prime \prime }$. That is, there are $m^{\prime \prime }-m^{\prime }$ packets are transmitted in this phase. The detailed procedure is given.

**S1.**  The FMCC, DMAM and MIGW decide to execute the data migration procedure
**S2.**  DMAM sends a session-migration-approved message, SMA_message, to FMCC. DMAM instructs FMCC to generate the essential traffic of the control plane by ensuring the seamless service migration procedure below. After FMCC receiving SMA_message from DMAM, it enables OpenFlow rules of the FMCC. FMCC sends out *session-migration-request* message, SMR_message to notify $c_{s}$ and $c_{d}$ to execute the data migration. Based on information of TIM message, all un-transmitted packets, for all $b_{k}=0$ and m+1≤k≤n, including TIM message are migrated from DC of $c_{s}$ to DC of $c_{d}$.
**S3.**  After DC of cloud $c_{d}$ verifying the received TIM message, the DC of cloud $c_{d}$ randomly pre-transmits some $k^{\prime }$-th un-transmitted packets, where $b_{k}^{\prime }=0$ and $m+1\leq k^{\prime }\leq n$. The corresponding bits are set 1, for $b_{k}^{\prime }=1$, in the TIM message if the packet are successfully pre-transmit toward to cloudlet $l_{s}$ and keep the pre-transmitted packets in cache of $l_{s}$. This pre-transmission operation is done until the new route path is determined in *follow-me cloud-cloudlet* phase.

**Figure 9:**
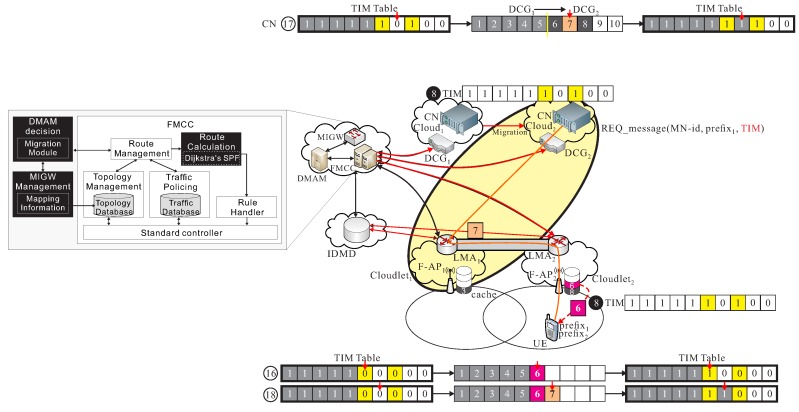
An example of *follow me cloud* phase.

As shown in Figure 9, 8-th packet is transmitted if TIM = (1, 1, 1, 1, 1, 1, 0, 1, 0, 0), and $b_{7}=0$, from the last phase. Finally, TIM = (1, 1, 1, 1, 1, 1, 1, 1, 0, 0), and the above operation is done before the new route path from $c_{d}$ to $l_{d}$.

### 4.4. Follow-Me Cloud-Cloudlet Phase

The phase mainly implements the cooperation of cloud-cloudlet $CL_{d\to d}$. We now have Then, we have $TIM=(\underset{m^{\prime \prime }}{\underset{︸}{1,1,\cdots ,1}}\underset{n-m^{\prime \prime }}{\underset{︸}{0,0,\cdots ,0}})$, where $m\leq m^{\prime \prime }$. After the new route path is re-routed from the new serving data center of $c_{d}$ to the new cloudlet $l_{d}$ which is determined by FMCC, $n-m^{\prime \prime }$ un-transmitted packets are transmitted from $c_{d}$ to UE through F-AP${}_{d}$ of $l_{d}$ by using the new re-calculated route path. The detailed procedure is given.

**S1.**  When a new route path from DC of $c_{d}$ to DC of $l_{d}$ is re-calculated in route calculation module of FMCC, FMCC generates the OpenFlow *flow-mod* message to DCG${}_{d}$ of $c_{d}$ to LMA${}_{d}$ of $l_{d}$, the new re-calculated route from DCG${}_{d}$ of $c_{d}$ to LMA${}_{d}$ of $l_{d}$ is then constructed.
**S2.**  All *k*-th un-transmitted packets from the final TIM message, for all if $b_{k}=0$ and m+1≤k≤n are sequentially transmitted by using the new re-calculated route from DCG${}_{d}$ of $c_{d}$ to LMA${}_{d}$ of $l_{d}$. Finally, all packets are successfully received by UE such that $TIM=TIM\_UE=(\underset{n}{\underset{︸}{1,1,\cdots ,1}})$.

**Figure 10:**
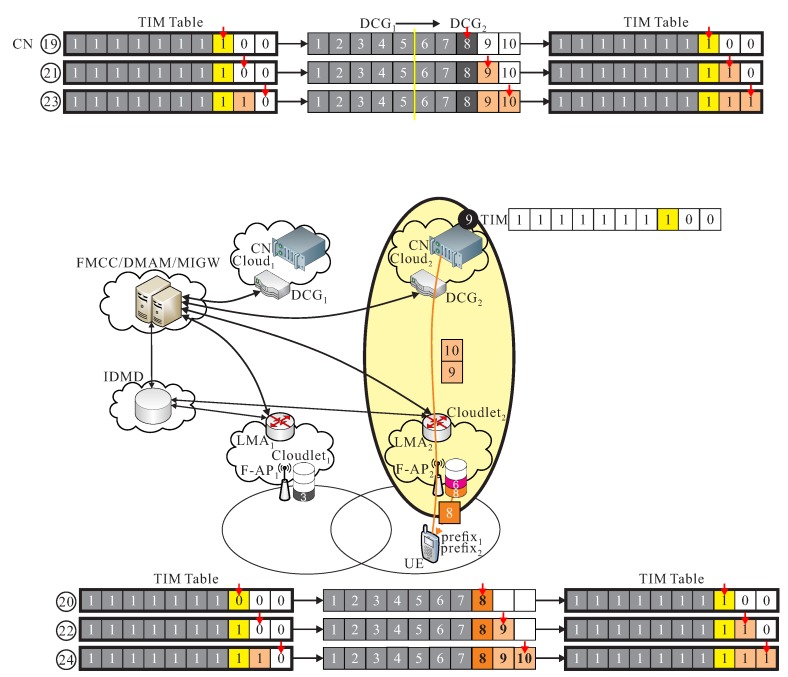
An example of *follow me cloud-cloudlet* phase.

As shown in Figure 10, since TIM = (1, 1, 1, 1, 1, 1, 1, 1, 0, 0), then 9-th and 10-th packets are transmitted from CN of $c_{d}$ and finally all packets are received, where TIM_UE = (1, 1, 1, 1, 1, 1, 1, 1, 1, 1).

## 5. Performance Analysis

Our paper presents a mobility management using FMCL approach F-RAN for smart cities. A mathematical analysis and simulation results are provided. Our paper presents a mobility management using FMCL approach. To evaluate our handover protocol (proposed scheme with FMCL), and Aissioui et al.’s. proxy MIPv6-based FMC (PMIPv6 with FMC) [24], all these protocols are mainly implemented using the Ryu and Mininet as illustrated in Table 1. Ryu [35] is a component-based software defined networking framework. Ryu provides software components with well defined API that make it easy for developers to create new network management and control applications. Ryu supports various protocols for managing network devices, such as OpenFlow, Netconf, OF-config, etc. About OpenFlow, Ryu supports fully 1.0, 1.2, 1.3, 1.4, 1.5 and Nicira Extensions. All of the code is freely available under the Apache 2.0 license [35]. Mininet [36] creates a realistic virtual network, running real kernel, switch and application code, on a single machine (VM, cloud or native). Mininet is a way to develop, share, and experiment with OpenFlow and Software-Defined Networking systems [36]. In our simulation, we built two two computers; one is installed a SDN controller, Ryu 4.1 (IP: 172.24.4.2); and another one is installed OpenFlow-based Software-Defined Networking systems by mininet (IP: 172.24.24.2) under the same IP domain.

Before describing the performance metrics, the mathematical analysis of the total transmission time and space cost is described.

**Theorem** **1.** The total transmission time of the proposed mobility management with FMCL approach is $T=\sum_{1\leq i\leq m}\left[\alpha t_{FM_{l}}\left(p_{i}\right)+\left(1-\alpha \right)t_{FM_{c}}\left(p_{i}\right)\right]+\sum_{m<i\leq m^{\prime }}t_{FML}\left(p_{i}\right)+\sum_{m^{\prime }<i\leq m^{\prime \prime }}t_{FMC}\left(p_{i}\right)+\sum_{m^{\prime \prime }<i\leq n}\left[\beta t_{FMCL_{l}}\left(p_{i}\right)+\left(1-\beta \right)t_{FMCL_{c}}\left(p_{i}\right)\right]+\sum_{m^{\prime }<i\leq n}t_{m}\left(p_{i}\right)$, where the cache hit rates of packets in caches of $l_{s}$ and $l_{d}$ are α and β, respectively.

**Figure 11:**
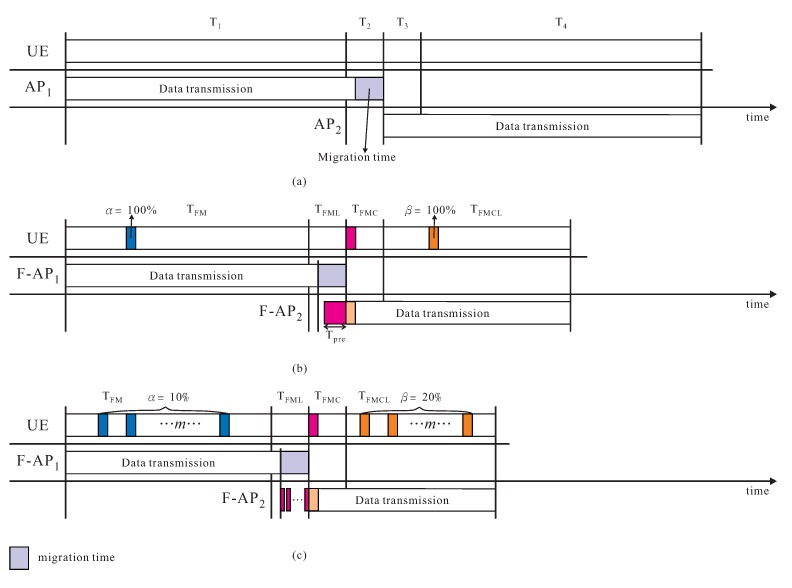
Example of the time and space costs of (**a**) proxy MIPv6-based FMC, and our proposal scheme with FMCL if (**b**) α=β=100%, and (**c**) α=10% and β=20%.

**Proof.**  Following notations in Section 4, a data file is divided into *n* packets, $F=\left\{p_{1},p_{2},\cdots ,p_{m},\cdots ,p_{m^{\prime }},\cdots ,p_{m^{\prime \prime }},\cdots ,p_{n}\right\}$, where $1\leq m<m^{\prime }<m^{\prime \prime }<n$. The total transmission time is divided into four phases, $T_{FM}$, $T_{FML}$, $T_{FMC}$, and $T_{FMCL}$ as illustrated in Figure 11a–c, where $T_{FM}$ is the time cost of performing *follow-me* phase, $T_{FML}$ is the time cost of performing *follow-me cloudlet* phase, $T_{FMC}$ denotes as the time cost of performing *follow-me cloud* phase, and $T_{FMCL}$ is the time cost of executing *follow-me cloud-cloudlet* phase. $T_{FM}=\sum_{1\leq i\leq m}\left[\alpha t_{FM_{l}}\left(p_{i}\right)+\left(1-\alpha \right)t_{FM_{c}}\left(p_{i}\right)\right]$, where the cache hit rate of packets in cache of $l_{s}$ is α, and $t_{FM_{l}}\left(p_{i}\right)$ and $t_{FM_{c}}\left(p_{i}\right)$ denote as the unit transmission time of packet $p_{i}$ transmitted from cloudlet $l_{s}$ or data center $c_{s}$, which is depended on packet $p_{i}$ ∈$l_{s}$ or $c_{s}$, where 1≤i≤m. The data migration time $T_{m}$ is $\sum_{m^{\prime }<i\leq n}t_{m}\left(p_{i}\right)$, where $t_{m}$ as the unit time of packet $p_{i}$ migrated from $c_{s}$ to $c_{d}$, where $m^{\prime }<i\leq n$. $T_{FML}=\sum_{m<i\leq m^{\prime }}t_{FML}\left(p_{i}\right)$, where $t_{FML}\left(p_{i}\right)$ is the unit transmission time of packet $p_{i}$ transmitted from data center $c_{s}$ to cloudlet $l_{d}$, where $m<i\leq m^{\prime }$. $T_{FMC}=\sum_{m^{\prime }<i\leq m^{\prime \prime }}t_{FMC}\left(p_{i}\right)$, where $t_{FMC}\left(p_{i}\right)$ is the unit transmission time of packet $p_{i}$ transmitted from data center $c_{d}$ to cloudlet $l_{s}$, where $m^{\prime }<i\leq m^{\prime \prime }$. $T_{FMCL}=\sum_{m^{\prime \prime }<i\leq n}\left[\beta t_{FMCL_{l}}\left(p_{i}\right)+\left(1-\beta \right)t_{FMCL_{c}}\left(p_{i}\right)\right]$, where the cache hit rate of packets in cache of $l_{d}$ is β, and $t_{FMCL_{l}}\left(p_{i}\right)$ and $t_{FMCL_{c}}\left(p_{i}\right)$ denote as the unit transmission time of packet $p_{i}$ transmitted from cloudlet $l_{d}$ or data center $c_{d}$, which is depended on packet $p_{i}$ ∈ $l_{d}$ or $c_{d}$, where $m^{\prime \prime }<i\leq n$. Consequently, the total transmission time $T=T_{FM}+T_{FML}+T_{FMC}+T_{FMCL}+T_{m}=\sum_{1\leq i\leq m}\left[\alpha t_{FM_{l}}\left(p_{i}\right)+\left(1-\alpha \right)t_{FM_{c}}\left(p_{i}\right)\right]+\sum_{m<i\leq m^{\prime }}t_{FML}\left(p_{i}\right)+\sum_{m^{\prime }<i\leq m^{\prime \prime }}t_{FMC}\left(p_{i}\right)+\sum_{m^{\prime \prime }<i\leq n}\left[\beta t_{FMCL_{l}}\left(p_{i}\right)+\left(1-\beta \right)t_{FMCL_{c}}\left(p_{i}\right)\right]+\sum_{m^{\prime }<i\leq n}t_{m}\left(p_{i}\right)$. ☐

**Theorem** **2.** The space cost of the proposed mobility management with FMCL approach is $\sum_{1\leq i\leq m,p_{i}\in l_{s}}S\left(p_{i}\right)$ + $\sum_{m^{\prime \prime }\leq i\leq n,p_{i}\in l_{d}}S\left(p_{d}\right)$, where $F=\left\{p_{1},p_{2},\cdots ,p_{m},\cdots ,p_{m^{\prime }},\cdots ,p_{m^{\prime \prime }},\cdots ,p_{n}\right\}$, $1\leq m<m^{\prime }<m^{\prime \prime }<n$, and $S(p_{i})$ denote as the space size of packet $p_{i}$.

**Proof.**  If $F=\left\{p_{1},p_{2},\cdots ,p_{m},\cdots ,p_{m^{\prime }},\cdots ,p_{m^{\prime \prime }},\cdots ,p_{n}\right\}$, and $1\leq m<m^{\prime }<m^{\prime \prime }<n$, as illustrated in Figure 11c, the space size of $l_{s}$ is $\sum_{1\leq i\leq m,p_{i}\in l_{s}}S\left(p_{i}\right)$ before the handover and the space size of $l_{d}$ is $\sum_{m^{\prime \prime }\leq i\leq n,p_{i}\in l_{d}}S\left(p_{d}\right)$ after the handover, the total space size is $\sum_{1\leq i\leq m,p_{i}\in l_{s}}S\left(p_{i}\right)$ + $\sum_{m^{\prime \prime }\leq i\leq n,p_{i}\in l_{d}}S\left(p_{d}\right)$. ☐

Example is offered in Figure 11a–c, the time and space costs of proxy MIPv6-based FMC is given in Figure 11a. The time and space costs of our proposal scheme with FMCL are given in Figure 11b–c if α=β=100% and if α=10% and β=20%, respectively. The performance metrics to be observed are:

- *Total transmission time* is the time cost of all *n* packets are successfully received by UE and transmitted from CN during the handover from F_AP${}_{l}$ to F_AP${}_{d}$, if CN has a data file $F=\left\{p_{1},p_{2},\cdots ,p_{n}\right\}$.
- *Throughput* is the total number of data packets that can be transmitted and received between UE and CN pair per unit time.
- *Probability of packet loss* is the total number of successfully received packets by UE divided by the total number of packets transmitted from CN.
- *Number of control messages* is the total number of control messages generated by the proposed mobility management using FMCL approach.

### 5.1. Total Transmission Time

**Figure 12:**
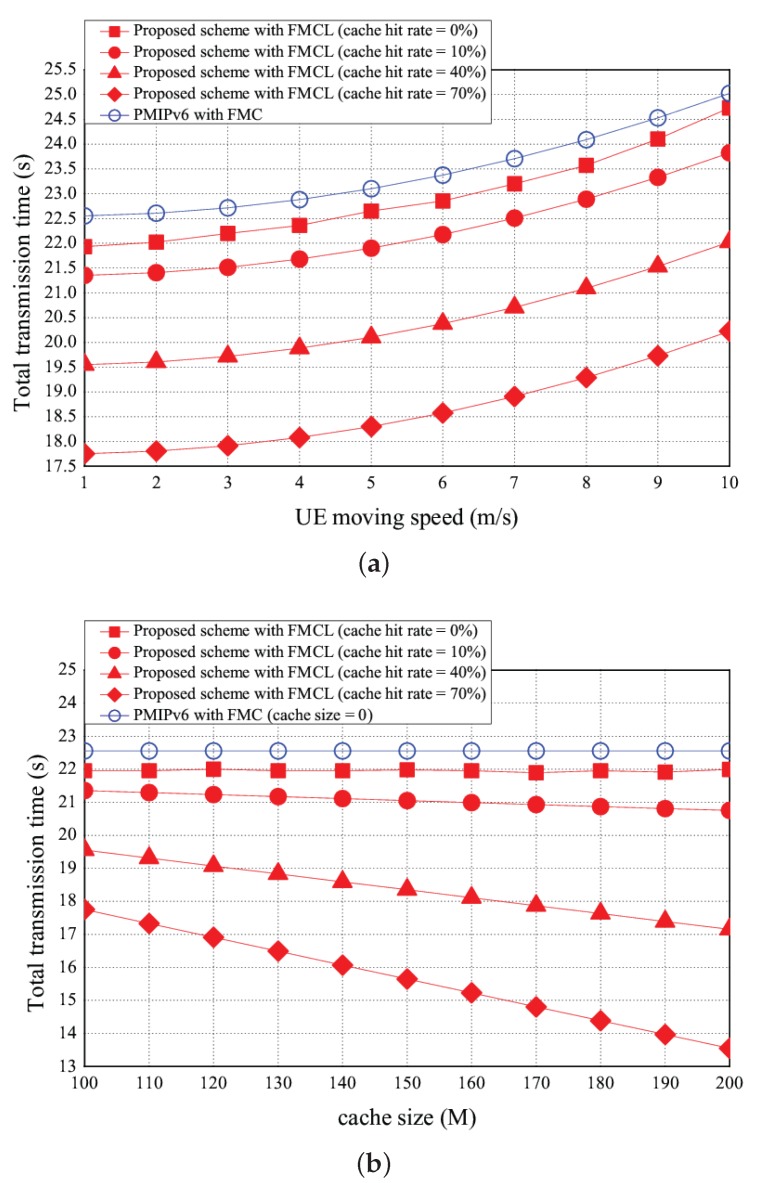

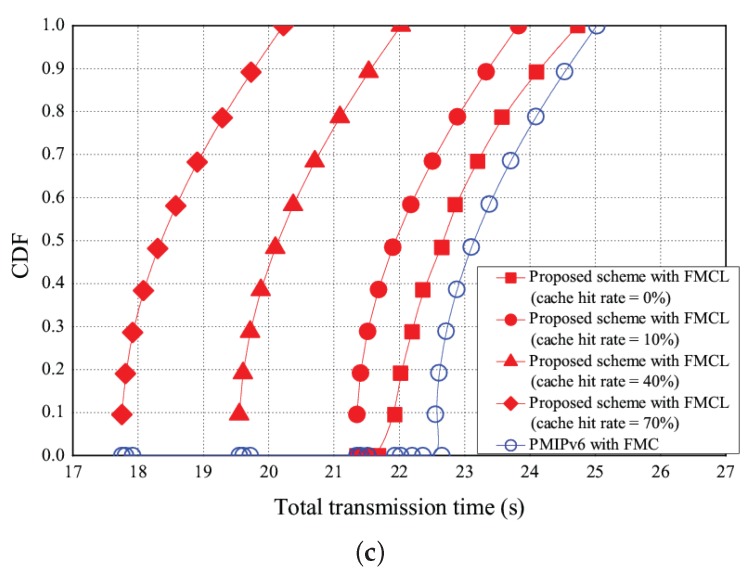
Total transmission time vs. (**a**) UE moving speed and (**b**) cache size, and (**c**) CDF vs. the total transmission time.

The simulation results of the total transmission time vs. the UE moving speed, the cache size, and CDF (cumulative distribution function) are shown in Figure 12a–c, respectively. As UE moving speed increases, the total transmission time reduces because that the handover frequency increases. The more number of handover requests is, the higher handover latency time will be. As shown in Figure 12a, the total transmission time can be reduced if the more packets can be discovered in the cache. For instance, Figure 12a shows that the total transmission time is 23 s using *PMIPv6 with FMC*. However, the total transmission time of our proposed scheme with FMCL are 22.6 s, 22 s, 20 s and 18 s if the cache hit rate is 0, 10%, 40%, and 70%, with the UE moving speed fixed at 5 m/s, respectively. Figure 12a shows that the total transmission time of PMIPv6 with FMC was > that of our proposed scheme with FMCL (the cache hit rate = 0%) was > that of our proposed scheme with FMCL (the cache hit rate = 10%) was > our proposed scheme with FMCL (the cache hit rate = 40%) was > our proposed scheme with FMCL (the cache hit rate = 70%) was, under the different UE moving speed. Figure 12b shows the total transmission time vs. the different size of the cache (is ranging from 100 M to 200 M). The higher the size of the cache is, the lower the total transmission time will be. This proposed scheme aims at developing a pre-transmission method to increase the number of packets in cache. For instance, Figure 12b shows that the total transmission time is 22.5 s using PMIPv6 with FMC if the cache size is 150 (M). However, the total transmission time of our proposed scheme with FMCL are 22 s, 21 s, 18.5 s and 15.6 s if the cache hit rate is 0, 10%, 40%, and 70%, if the cache size is 150 (M). Similarly, Figure 12b shows that the total transmission time of PMIPv6 with FMC was > that of our proposed scheme with FMCL (the cache hit rate = 0%) was > that of our proposed scheme with FMCL (the cache hit rate = 10%) was > our proposed scheme with FMCL (the cache hit rate = 40%) was > our proposed scheme with FMCL (the cache hit rate = 70%) was, under the different cache size. In addition, Figure 12c offers the result of CDFvs. the total transmission time.

### 5.2. Throughput

**Figure 13:**
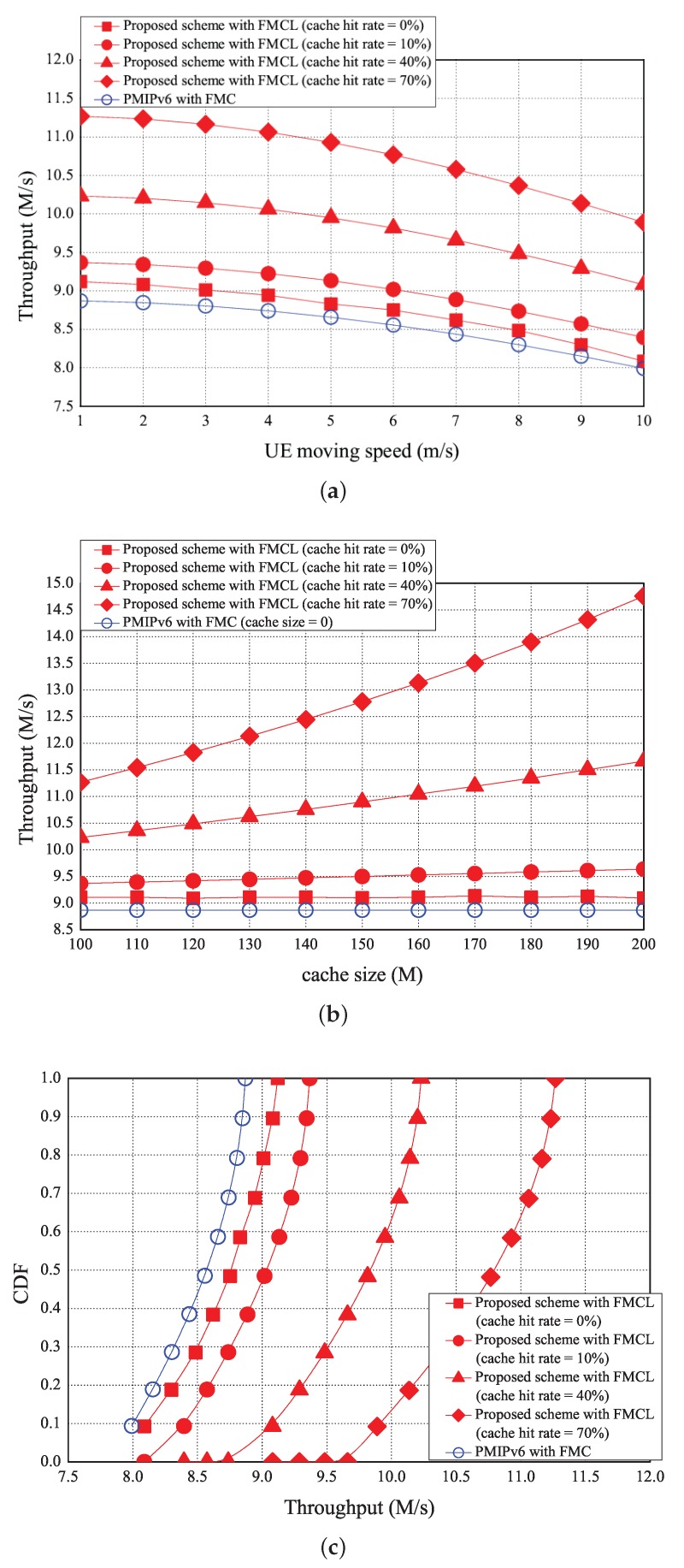
Throughput vs. (**a**) UE moving speed. and (**b**) cache size, and (**c**) CDF vs. throughput.

The simulation results of the average throughput vs. the UE moving speed, the cache size, and its CDF are illustrated in Figure 13a–c, respectively. On average, as UE moving speed increases, the average throughput decrease. As shown in Figure 13a, the usage probability of packets in cache will be decreased if the UE moving speed increases, it implies that the average throughput will be decreases. Figure 13a shows that the average throughput is 8.6 (M/s) using the PMIPv6 with FMC under the UE moving speed is 5 (m/s). However, the average throughput of our proposed scheme with FMCL are 8.8 (M/s), 9 (M/s), 10 (M/s), and 11 (M/s) if the cache hit rate is 0, 10%, 40%, and 70%, under the UE moving speed is 5 m/s, respectively. Figure 13a shows that the average throughput of PMIPv6 with FMC was < that of our proposed scheme with FMCL (the cache hit rate = 0%) was < that of our proposed scheme with FMCL (the cache hit rate = 10%) was < our proposed scheme with FMCL (the cache hit rate = 40%) was < our proposed scheme with FMCL (the cache hit rate = 70%) was, under the different UE moving speed. As shown in Figure 13b, the simulation result of the average throughput vs. the different cache size (is ranging from 100 M to 200 M). As the higher the size of the cache is, the higher the average throughput will be. Figure 13b shows that the average throughput is 8.9 (M/s) using the PMIPv6 with FMC under the UE moving speed is 5 (m/s). However, the average throughput of our proposed scheme with FMCL are 9.1 (M/s), 9.5 (M/s), 10.9 (M/s), and 12.8 (M/s) if the cache hit rate is 0, 10%, 40%, and 70%, under the cache size is 150 (M), respectively. Figure 13b also shows that the average throughput of PMIPv6 with FMC was < that of our proposed scheme with FMCL (the cache hit rate = 0%) was < that of our proposed scheme with FMCL (the cache hit rate = 10%) was < our proposed scheme with FMCL (the cache hit rate = 40%) was < our proposed scheme with FMCL (the cache hit rate = 70%) was, under the different cache size. In addition, the CDF vs. the average throughput is provided in Figure 13c.

### 5.3. Probability of Packet Loss

**Figure 14:**
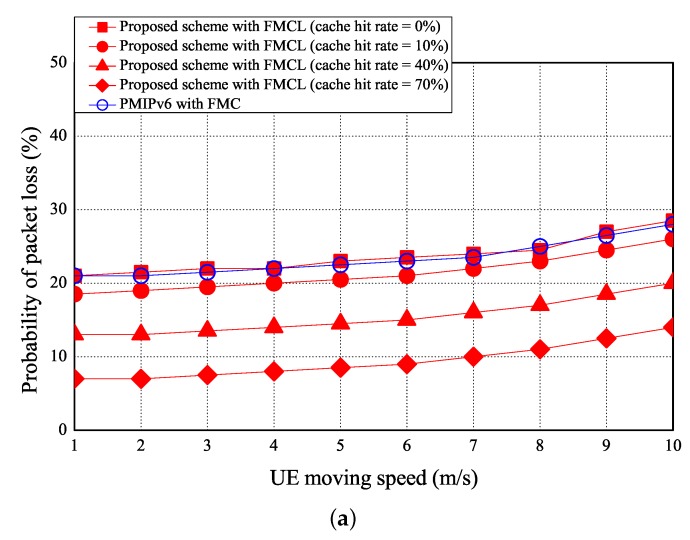

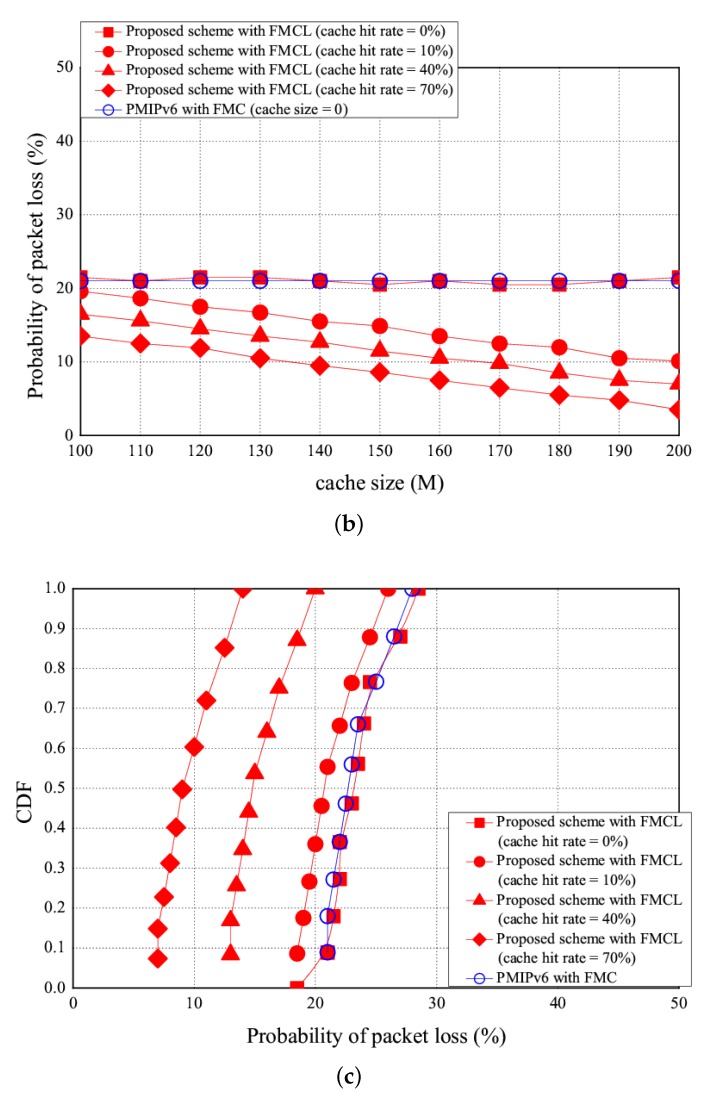
Probability of packet loss vs. (**a**) UE moving speed and (**b**) cache size, and (**c**) CDF vs. probability of packet loss.

The simulation results of the probability of packet loss v. the UE moving speed, cache size, and CDF are shown in Figure 14a–c, respectively. As the UE moving speed increases, the probability of packet loss reduces. As shown in Figure 14a, the probability of packet loss can be reduced if the more number of handover events occurred. Figure 14a shows that the probability of packet loss is 22.5 (%) using the PMIPv6 with FMC under the case of the UE moving speed is 5 (m/s). However, the probability of packet loss of our proposed scheme with FMCL are 23 (%), 20.5 (%), 14.5 (%), and 8.5 (%) if the cache hit rate is 0, 10%, 40%, and 70%, under the case of the UE moving speed is 5 m/s, respectively. Figure 14a shows that the probability of packet loss of PMIPv6 with FMC was ≈ that of our proposed scheme with FMCL (the cache hit rate = 0%) was > that of our proposed scheme with FMCL (the cache hit rate = 10%) was > our proposed scheme with FMCL (the cache hit rate = 40%) was > our proposed scheme with FMCL (the cache hit rate = 70%) was, under the different UE moving speed. Figure 14b shows that the simulation result of the probability of packet loss vs. the different cache size (is ranging from 100 M to 200 M). The higher the size of the cache is, the lower probability of packet loss will be. Figure 14b shows that the probability of packet loss is 21 (%) using the PMIPv6 with FMC under the case of the cache size is 150 (M) . However, the probability of packet loss of our proposed scheme with FMCL are 20.5 (%), 15 (%), 11.5 (%), and 8.6 (%) if the cache hit rate is 0, 10%, 40%, and 70%, under the case of the cache size is 150 (M), respectively. Figure 14b also shows that the probability of packet loss of PMIPv6 with FMC was ≈ that of our proposed scheme with FMCL (the cache hit rate = 0%) was > that of our proposed scheme with FMCL (the cache hit rate = 10%) was > our proposed scheme with FMCL (the cache hit rate = 40%) was > our proposed scheme with FMCL (the cache hit rate = 70%) was, under the different cache size. Finally, Figure 14c offers the result of the CDF vs. probability of packet loss.

### 5.4. Number of Control Messages

**Figure 15:**
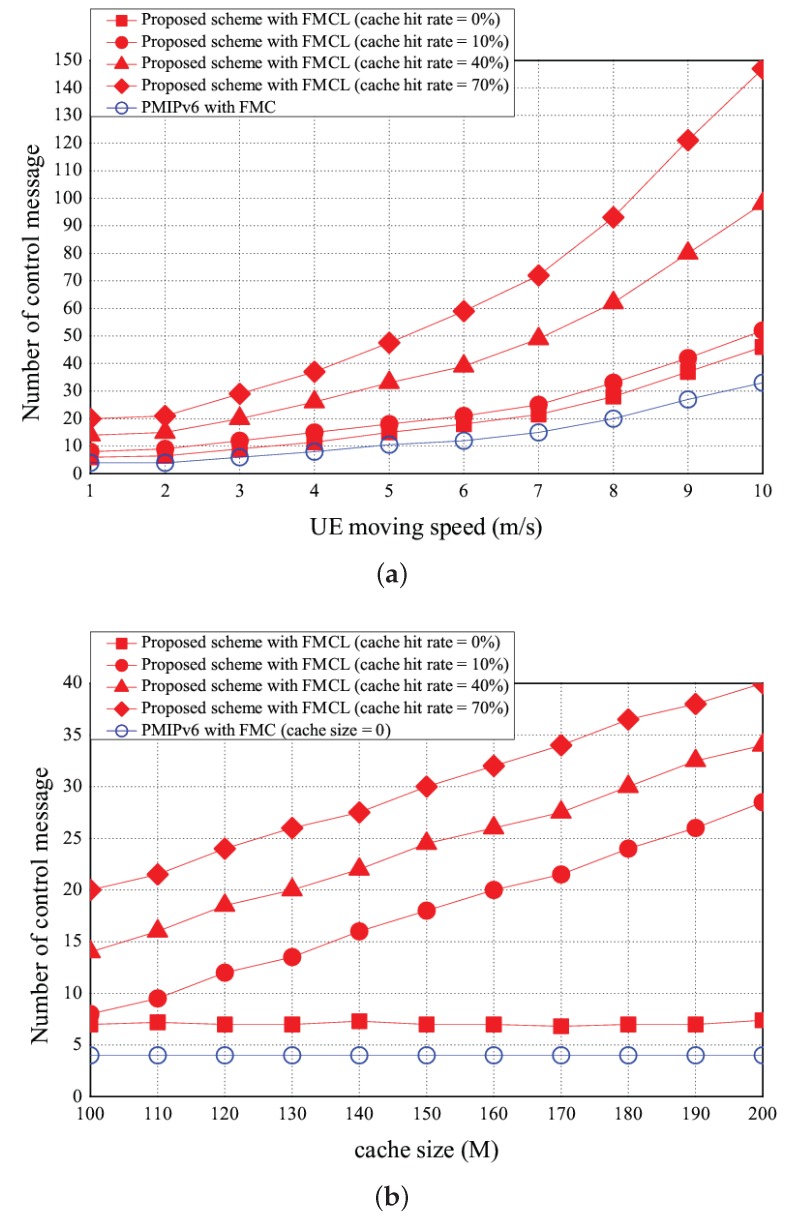

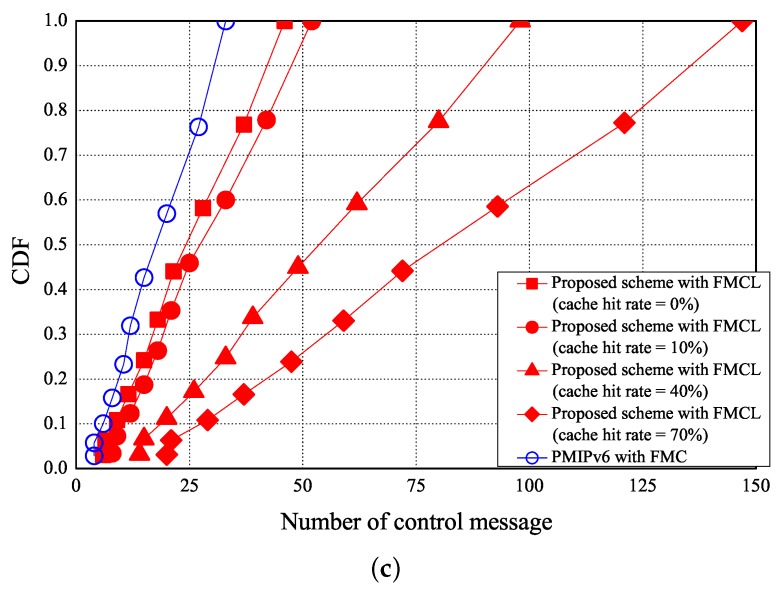
Number of control messages vs. (**a**) UE moving speed and (**b**) cache size, and (**c**) CDF vs. number of control messages.

The simulation results of the number of control message vs. the UE moving speed, cache size, and CDF are shown in Figure 15a–c, respectively. As shown in Figure 15a, the the number of control message increases if the more number of handover events occurred. Figure 15a shows that the number of control messages is 10 using the PMIPv6 with FMC under the case of the UE moving speed is 5 (m/s). However, the number of control messages of our proposed scheme with FMCL are 15, 18, 33, and 48 if the cache hit rate is 0, 10%, 40%, and 70%, under the case of the UE moving speed is 5 m/s, respectively. Figure 15a shows that the number of control messages of PMIPv6 with FMC was < that of our proposed scheme with FMCL (the cache hit rate = 0%) was < that of our proposed scheme with FMCL (the cache hit rate = 10%) was < our proposed scheme with FMCL (the cache hit rate = 40%) was < our proposed scheme with FMCL (the cache hit rate = 70%) was, under the different UE moving speed. Figure 15b shows simulation result of the number of control messages vs. the different cache size (is ranging from 100 M to 200 M). The higher cache size is, the higher the number of control message will be. This is because that as the high cache size also implies that the high cache hit rate, so more control messages to needed to communicate with UE and F_AP. Figure 15b shows that the number of control messages is 4 using the PMIPv6 with FMC under the case of the cache size is 150 (M). However, the probability of packet loss of our proposed scheme with FMCL are 7, 18, 24, and 30 if the cache hit rate is 0, 10%, 40%, and 70%, under the case of the cache size is 150 (M), respectively. Figure 15b illustrates that the the number of control messages of PMIPv6 with FMC was < that of our proposed scheme with FMCL (the cache hit rate = 0%) was < that of our proposed scheme with FMCL (the cache hit rate = 10%) was < our proposed scheme with FMCL (the cache hit rate = 40%) was < our proposed scheme with FMCL (the cache hit rate = 70%) was, under the different cache size. Finally, Figure 15c provides the simulation result of CDF vs. the number of control messages.

## 6. Conclusions

This paper is to propose a new mobility management by using a *follow-me cloud-cloudlet* (FMCL) approach, which is a cooperation with cloud and cloudlet in Fog-RANs. All existing FMC results do not have the property of the VM migration between cloudlets, and all existing FME results do not have property of the service migration between data centers (DCs). As we can known, it is the first result to report a hybrid scheme by combining FMC and FME schemes into a fully new FMCL approach. The main contribution of the proposed mobility management using FMCL approach is to simultaneously keep the VM migration between cloudlets and service migration between DCs to significantly reduce the transmission latency in Fog-RANs. In addition, our mathematical analysis and simulation results shows that our proposed mobility scheme with FMCL approach outperforms existing FMC result in terms of the total transmission time, the average throughput, and the probability of packet loss, but with the more number of the control messages.

Future work can be further considered the multicast problem using the new proposed FMCL approach in fog-computing-based RAN for smart cities.

## Figures and Tables

**Table 1:** Simulation environment and parameters.

Parameter	Value
Simulation tools	Ryu SDN frame-work Mininet
Bandwidth per link	10 Mbps
Data file size	200 M
Number of F-AP	2
Number of DC	2
Number of UEs	0 to 50
Packet size	Uniform distribution with min = 84, max = 500 bytes
